# Measuring, manipulating and exploiting behaviours of adult mosquitoes to optimise malaria vector control impact

**DOI:** 10.1136/bmjgh-2016-000212

**Published:** 2017-04-26

**Authors:** Gerry F Killeen, John M Marshall, Samson S Kiware, Andy B South, Lucy S Tusting, Prosper P Chaki, Nicodem J Govella

**Affiliations:** 1 Environmental Health and Ecological Sciences Department, Ifakara Health Institute, Ifakara and Dar es Salaam, United Republic of Tanzania; 2 Department of Vector Biology, Liverpool School of Tropical Medicine, Liverpool, UK; 3 Divisions of Biostatistics and Epidemiology, School of Public Health, University of California, Berkeley, California, USA; 4 Norwich, UK; 5 Oxford Big Data Institute, Li Ka Shing Centre for Health Information and Discovery, University of Oxford, Oxford, UK

## Abstract

Residual malaria transmission can persist despite high coverage with effective long-lasting insecticidal nets (LLINs) and/or indoor residual spraying (IRS), because many vector mosquitoes evade them by feeding on animals, feeding outdoors, resting outdoors or rapidly exiting from houses after entering them. However, many of these behaviours that render vectors resilient to control with IRS and LLINs also make them vulnerable to some emerging new alternative interventions. Furthermore, vector control measures targeting preferred behaviours of mosquitoes often force them to express previously rare alternative behaviours, which can then be targeted with these complementary new interventions. For example, deployment of LLINs against vectors that historically fed predominantly indoors on humans typically results in persisting transmission by residual populations that survive by feeding outdoors on humans and animals, where they may then be targeted with vapour-phase insecticides and veterinary insecticides, respectively. So while the ability of mosquitoes to express alternative behaviours limits the impact of LLINs and IRS, it also creates measurable and unprecedented opportunities for deploying complementary additional approaches that would otherwise be ineffective. Now that more diverse vector control methods are finally becoming available, well-established entomological field techniques for surveying adult mosquito behaviours should be fully exploited by national malaria control programmes, to rationally and adaptively map out new opportunities for their effective deployment.

**Key questions**

**What is already known about this topic?**

- Specific mosquito behaviours, such as outdoor resting, outdoor feeding, feeding on animals and early exiting from houses, allow malaria vectors to avoid exposure to insecticides delivered to houses in the forms of long-lasting insecticidal nets (LLINs) and/or indoor residual sprays (IRS).
- Mosquitoes which exhibit one or more of these behaviours are responsible for persistent residual malaria transmission, even where high coverage of LLINs and/or IRS has been achieved.

**What are the new findings?**

- While these behaviours make mosquito populations robust to control with LLINs and IRS, they also make them vulnerable to emerging new vector control technologies that target them while feeding outdoors on humans or cattle.
- Scaling up interventions that selectively target any specific blood feeding behaviour increases the proportional contributions of alternative behaviours to mosquito survival, so that these can then be targeted with complementary additional interventions. For example, following a scale-up of LLINs to target indoor-feeding mosquitoes, surviving mosquitoes obtain most of their blood meals outdoors from humans and livestock, where they may be targeted with insecticidal clothing or vapour emanators and veterinary insecticides, respectively.

**Recommendations for policy**

- National malaria control programmes should now take full advantage of long-established, practical and affordable entomological field survey methods, to identify, create and exploit opportunities for effectively targeting adult mosquitoes with a greater diversity of control measures.
- The creative, adaptive, problem-solving traditions of the discipline once known as *epidemiological entomology* need to be urgently revived and rejuvenated at all levels of policy and practice.

## Introduction

Malaria vector control with long-lasting insecticidal nets (LLINs) or indoor residual spraying (IRS) has been remarkably successful over recent years, accounting for most of the 663 million cases and 4 million deaths averted since 2000.^1^ ^2^ LLINs and IRS have been most effective in regions of high transmission where local vectors like *Anopheles funestus* and *A. gambiae* in Africa, or *A. punctulatus* and *A. koliensis* in the Pacific, exhibit human-feeding, indoor-feeding and indoor-resting behaviours that are vulnerable to attack with LLINs and/or IRS.^3–5^ However, LLINs and IRS are poorly suited to tackling the much larger number of important vector species that avoid fatal contact with these products by feeding outdoors, by frequently feeding on animals, resting outdoors or foraging briefly and cautiously within houses when they do enter them.^3^ ^5^ ^6^ Thus, for many high-risk populations, elimination of *residual malaria transmission* is unattainable, even with full universal coverage of highly effective LLINs and/or IRS, using active ingredients to which the local vectors are fully susceptible.^3^ ^6^ ^7^

However, a number of rejuvenated, repurposed and entirely new vector control methods are now emerging that can address residual malaria transmission by complementing, and even superseding, current LLIN and IRS technologies.^8^ It is therefore time to be more optimistic, and urgently rethink how we look at malaria vector behaviours. Specifically, we need to start viewing phenomena like outdoor feeding, feeding on animals and early exit from houses as missed opportunities for rational deployment of new interventions, rather than merely obstacles to success with existing IRS and LLIN options.

### Turning problems into opportunities

Fortunately, many behaviours that render vectors resilient to IRS and LLINs also make them vulnerable to emerging new alternatives. New or improved vector control strategies for dealing with residual transmission are now emerging that exploit specific, quantifiable, vulnerable behaviours of adult mosquitoes, the first three of which were previously viewed as problems rather than potential solutions: (1) exclude, repel or kill adult vectors attempting to feed or rest inside houses; (2) repel, incapacitate or even kill adult mosquitoes when they attack people outdoors; (3) kill adult mosquitoes when they attack livestock; or (4) kill adult mosquitoes when they feed on sugar; (5) kill adult mosquitoes when they aggregate as mating swarms within human settlements.^8^

Taking the example of mosquitoes like *A. arabiensis* or *A. darlingi*, which can persistently forage indoors despite high coverage of LLINs or IRS^9^ ^10^ by avoiding extended contact with treated surfaces,^11–14^ this frustrating behavioural ability also provides convenient opportunities for preventing them from entering houses with traditional screening methods.^15^ Being more ambitious, it should even be possible to deliberately target them when they attempt to enter houses, with either entry traps^16^ or improved insecticides delivery formats.^17–19^

Similarly, where vectors like *A. farauti* or *A. epiroticus* frequently attack people while they are active outdoors, this can be viewed as an unexploited opportunity to target them by protecting humans with insecticide-treated clothing,^20^ ^21^ or new, long-lasting vapour emanator formulations of volatile insecticides^22–24^ that can debilitate^25^ or even kill^26^ vectors. Even vectors like *A. arabiensis*, which can feed often enough on humans to mediate intense transmission but extensively enough on cattle to be resilient against attack with IRS, LLINs or any other insecticidal personal protection measure for humans,^27^ may be tackled by deliberately targeting insecticides to these alternative blood sources. Where zoophagy predominantly results in frequent feeding on livestock rather than wild animals, veterinary formulations of topical or systemic insecticides (the latter are often referred to as endectocides) may be deployed, which are far more affordable, acceptable and long-lasting than available formulations of the same active ingredients for humans, through delivery systems that already exist in many low-income countries.^28^

### Manipulating vector behaviours to create new intervention opportunities

Furthermore, previously unusual behaviours of adult mosquitoes often become vital to their continued survival following deployment of interventions targeting more common behaviours, creating measurable new opportunities for complementary additional approaches to target these engineered vulnerabilities.

**Figure 1:**
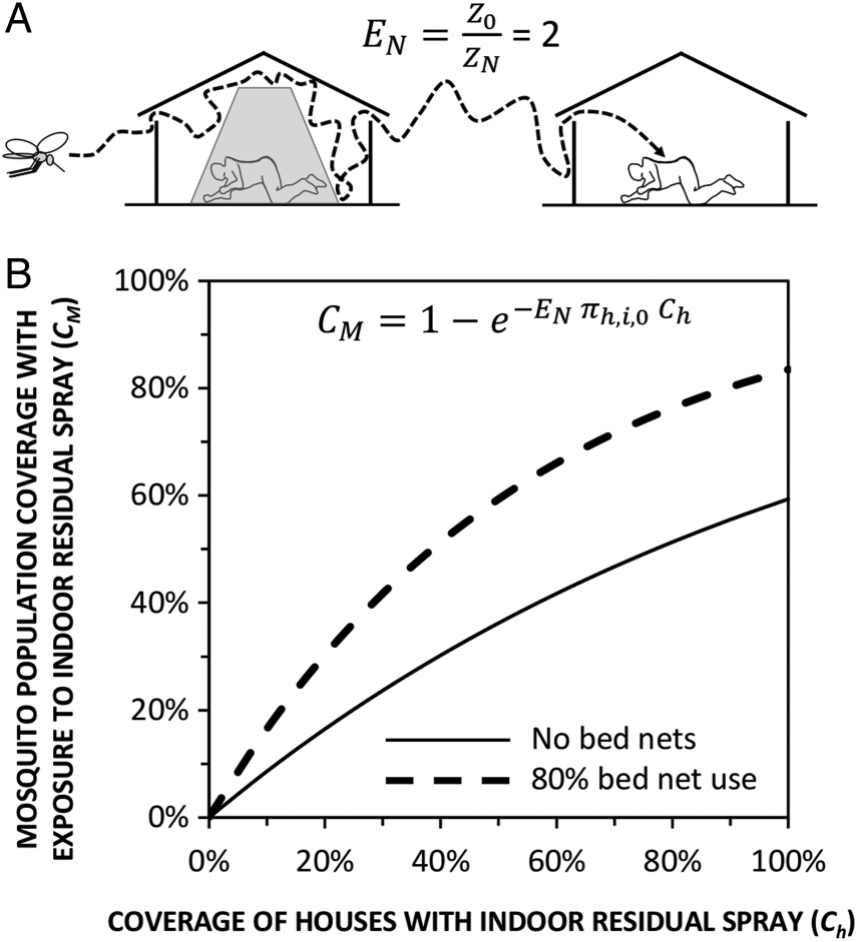
An illustration of how high coverage with bed nets can enhance the impact of a second domestic vector control measure with insecticides, such as IRS, by forcing mosquitoes to visit far more houses than they normally would. (A) A schematic representation of how reducing the availability of human blood (Z) with 80% human usage (U_h_=0.8) of bed nets (N) can double the number of encounters (E) with humans required by *Anopheles arabiensis* to obtain a blood meal, relative to baseline conditions with no nets (0).^10^ (B) Estimated coverage of the mosquito population (C_M_) with exposure to insecticide^28^ delivered through IRS, at varying levels of house coverage (C_h_). Mosquito population coverage is expressed as the proportion of mosquitoes exposed to insecticide per feeding cycle and calculated by expressing equation 8 of a previously published model^28^ using the same notation as the model of *A. arabiensis* early-exit behaviour,^10^ assuming that 90% of all attacks on humans would occur indoors in the absence of any protection measure (π_h,i,0_=0.9). IRS, indoor residual spraying.

For example, in an east African setting with which we are particularly familiar, *A. arabiensis* historically fed predominantly indoors on humans despite their preference for cattle, because at that time cattle were scarce while people were both abundant and unprotected.^29^ ^30^ Following scale-up of LLINs, anthropophagic *A. funestus* became far more scarce and *A. gambiae* almost disappeared but *A. arabiensis* persisted^31^ ^32^ by exhibiting three behaviours which protect it against LLINs, as well as render it remarkably vulnerable to complementary measures: (1) increased feeding outdoors in the early evenings when people are active and unprotected by nets,^32^ where they could now be targeted with insecticide-treated clothing^20^ ^21^ or vapour-phase insecticides;^22–24^ (2) although they avoid fatal contact with LLINs when they do enter houses,^12^ ^13^ the fact is that bed nets force mosquitoes to enter twice as many houses to obtain the blood they need.^10^ This phenomenon of repeated house entry could therefore be exploited to kill them more effectively than would otherwise be possible, by applying additional insecticides inside houses by spraying them on the walls as IRS (figure 1), or by targeting them to entry points with eave tubes^19^ or exit points with eave baffles^17^; and (3) half of their blood meals are now obtained from unprotected cattle^34^ that do not use nets but could be readily treated with long-lasting veterinary insecticide formulations.^28^

**Figure 2:**
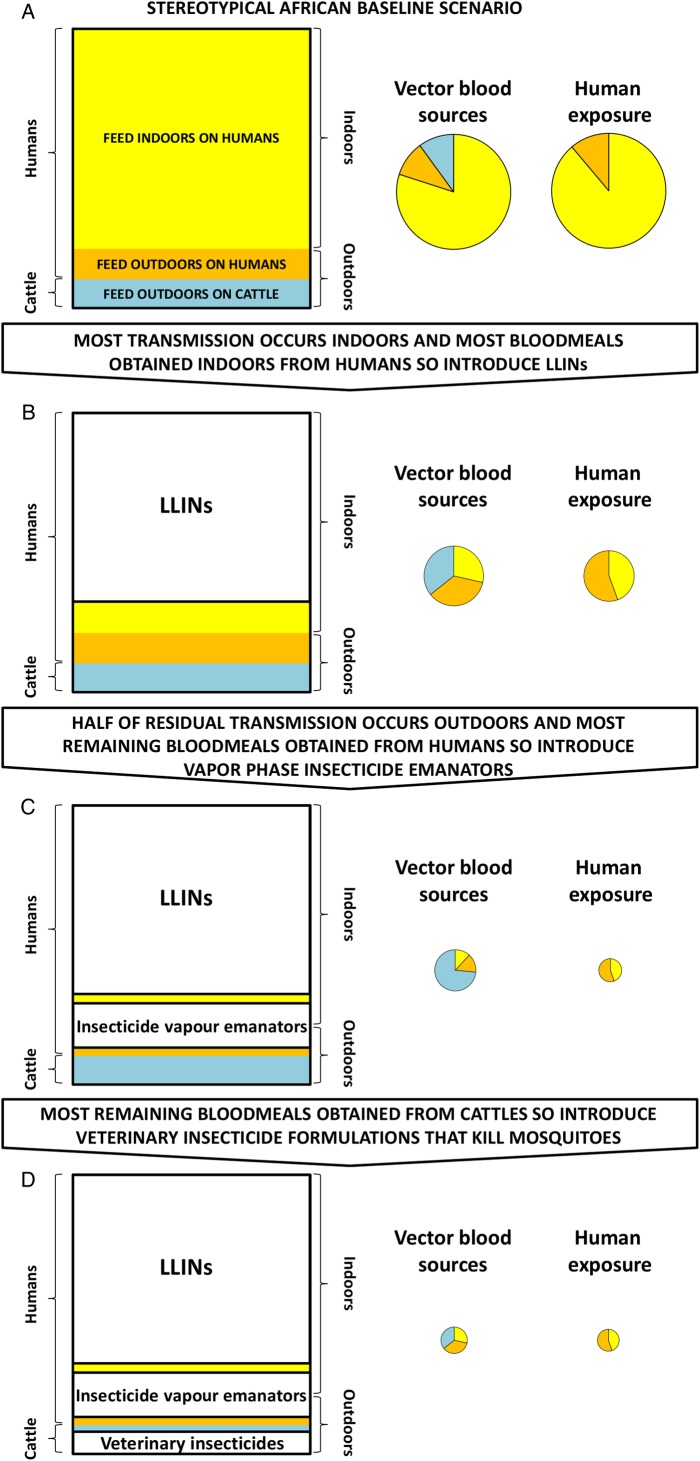
A schematic illustration of how sequential layers of vector control interventions against particular fractions of blood-feeding mosquitoes can create measurable opportunities for complementary approaches to achieve increasingly dramatic impacts on vector survival and residual transmission. This illustration is based on the well-characterised example of *Anopheles arabiensis* in southern Tanzania,^10^ as described in the section entitled *Manipulating vector behaviours to create new intervention opportunities*. We provide a simple online interactive graphical model (https://andysouth.shinyapps.io/coverage1/) allowing the reader to investigate the implications of combining interventions targeting different behaviour patterns under different baseline scenarios of proportional feeding indoor and on humans. The source code (in the statistical language R) is also provided so that the reader can run offline (https://github.com/AndySouth/coverage). LLIN, long-lasting insecticidal net.

As illustrated mechanistically in figure 2, such layering of interventions in a logical sequence can enable rational manipulation and exploitation of mosquito behaviour patterns, sometimes referred to as ‘push–pull’ strategies^35–38^ that originate from the agriculture sector.^39^ Such altered postintervention behavioural patterns create new opportunities for targeting outdoor-feeding vectors with insecticide clothing treatments,^18^ ^19^ insecticide vapour emanators^22–24^ and/or veterinary insecticides^28^ that would previously have been ineffective. These intervention options can therefore be expected to have a much greater impact on residual transmission as second-line and third-line measures to supplement LLINs in an elimination programme than they ever could on baseline transmission as the first-line measure in a control programme. Interestingly, a similar rationale may even be applicable to enhance the impact of attractive toxic sugar baits,^40^ because mosquitoes with less access to blood often maintain their increased energetic requirements by consuming more sugar.

As each layer of intervention tackles the fraction of transmission it is best suited to, the altered characteristics of the remaining residual transmission should be reassessed entomologically, to identify the new opportunities that emerge as the remaining fractions become progressively more vulnerable to well-chosen complementary strategies. Continuous, or at least regular, remeasurement of these behavioural metrics for monitoring purposes is essential because the heritable behavioural preferences of vector populations can change in response to selection pressure exerted by selectively targeted interventions.^41^ ^42^ Beyond the simple, instantaneous plasticity assumed in figure 2 that can be described as behavioural *resilience*, mosquitoes can also evolve behavioural *resistance* in the true sense,^42^ exhibiting altered patterns of innate feeding preferences over the longer term.^43^ ^44^

The observations of highly plastic blood-feeding behaviours by *A. arabiensis* in southern Tanzania, as described above, represent neither an isolated example nor a new paradigm, and figure 2 could well be described as a ‘glass-half-full’ reinterpretation of our previous simulations of these same behavioural processes.^42^ Indeed, this narrative for our local *A. arabiensis* population is just one out of hundreds of similar historical and contemporary examples reported for numerous vector species all across the tropics.^5^ ^45–49^ In fact, even the more anthropophagic African species *A. coluzzi*, *A. gambiae* and *A. funestus* have recently been observed to persist following LLIN/IRS scale-up by switching to feeding on animals.^50^ ^51^

### Exploiting the full potential of existing entomological field techniques

Most of the survey methods required to measure mosquito behaviours and enable optimal intervention selection (table 1) have been available for decades in practical low-technology formats that are accessible and affordable to national control and elimination programmes. While much more advanced laboratory techniques are now available for identifying which hosts mosquitoes obtain blood from,^52^ ^53^ the current state of the art for representatively sampling blood-fed mosquitoes in the field^49^ largely derives from classical texts.^45^ ^46^ New field techniques now extend the applicability of these approaches by making it possible to capture fed specimens of outdoor-resting species, which could not previously be obtained because they were too widely scattered across expansive outdoor-resting site habitats.^56^ Similarly, recent adjustments of mosquito biting rate measurements to account for human behaviours when estimating the distributions of where and when they are actually exposed to bites^3^ ^57–63^ are not entirely new: Similar exposure distribution graphs were occasionally used historically, back in the era of the Global Malaria Eradication Programme (GMEP).^11^ ^64^ ^65^ While the greatest obstacle to such measurements has been reliance on the notoriously hazardous human landing catch,^66^ recent evaluations of customised electric grid traps^67^ suggest that an end to this controversial and archaic field technique may be in sight. Perhaps the simplest of all targetable behaviours to measure is sugar feeding, requiring only the substitution of insecticide with food dye in attractive sugar baits, and a variety of well-established insect labelling methods exist that could be deployed to measure contact or usage rates for other targetable behaviours, including aggregation into mating swarms.^52^

**Table 1:** Opportunity indicators, niches and challenges for available and emerging vector control technologies targeting adult malaria vectors

Technology	Human indicator	Entomological indicator	Niche	Challenges
Physical mosquito proofing of				
Residential housing	At least partially sedentary lifestyles and sleep indoors	At least one-third of historical or current human exposure to vectors occurs indoors	Almost ubiquitous	Establish systems for promotion and subsidisation of affordable materials
Temporary or mobile shelters	At least partially migrant lifestyles and sleep in shelters		Almost ubiquitous	Develop locally appropriate, affordable prototype products
Traps or insecticide-treated window screens, eave tubes or eave baffles for killing mosquitoes attempting to enter houses or shelters	Sleep indoors or inside shelters	At least one-third of historical or current human exposure to vectors occurs indoors and at least one-third of blood meals are obtained from humans	Almost ubiquitous	Establish systems for promotion and subsidisation of affordable materials, including insecticide retreatments Develop locally appropriate, affordable prototype products
Insecticide-treated clothing or emanators for vapour-phase repellent, incapacitant and/or lethal insecticide	Outdoor activities common during hours of darkness	At least one-third of current human exposure to vectors occurs outdoors	Almost ubiquitous	Reformulation of volatile pyrethroids to maximise affordability, durability and safety Development of products with non-pyrethroid active ingredients
Insecticide treatments for livestock	Livestock ownership	At least one-third of vector blood meals are obtained from identified livestock species	Almost ubiquitous	Identify products which most effectively perform both their primary veterinary function and kill locally important malaria vectors
Insecticidal sugar baits	None known	Most vectors can be labelled with dyed baits lacking insecticide or killed by baits including insecticide	Unknown	Identify best available products and bespoke prototypes Map out geographic extent and variability of high sugar feeding rates and corresponding potential for impact Identify consistently optimal environmental targets and delivery strategies Demonstrate lack of environmental impact on non-target species of arthropods, pollinators in particular
Insecticidal aerosols or fogs targeted at mosquitoes when they disperse, rest or form mating swarms	None known	Most vectors can be labelled by dyed with formulations lacking insecticide or killed by formulations including insecticide	Possibly west and central Africa	Identify best available products and bespoke prototypes Map out geographic extent and variability of swarming within human settlement and corresponding potential for impact Identify consistently optimal environmental targets and delivery strategies Demonstrate lack of environmental impact on non-target species of arthropods

The indicators, prioritisation threshold values and niches for application of these vector control technologies are synthesised from a previous detailed review and modelling analyses.^3^ ^9^ ^10^ ^52–55^ For referenced discussion of the methodology required to survey each indicator, see the first paragraph of the section entitled *Exploiting the full potential of existing entomological field techniques*.

**Figure 3:**
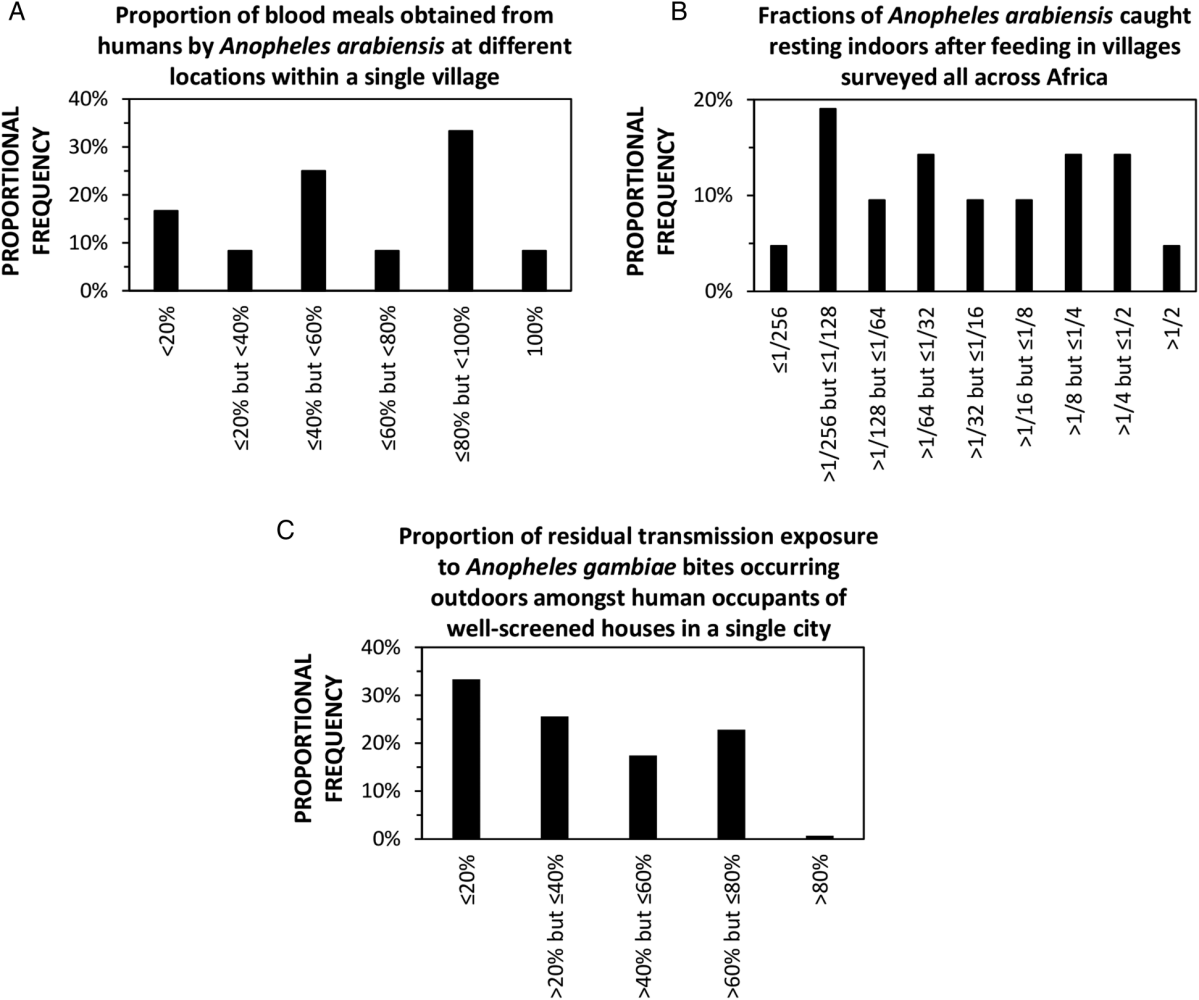
Examples of extremely heterogeneous behavioural outcomes, which arise from behavioural plasticity of malaria vector mosquitoes and their human victims, and occur across the full range of spatial scales that are relevant to vector control intervention selection. (A) Specimens of blood-fed, indoor-resting *Anopheles arabiensis* sampled from 12 different locations within a single village in northern Tanzania yielded estimates for the proportion of blood meals obtained from humans,^68^ which are distributed across the full possible range of values. (B) The estimated fraction of *A. arabiensis* which rest indoors after feeding (reported originally as the estimated usage rate for indoor resting sites per feeding cycle^52^) varies across a range of more than 300-fold in 21 distinct villages surveyed all across Africa. (C) Variations of only 1–3 hours in the times at which people go indoors for the evening and leave the house in the morning, among 9458 occupants of houses with well-screened windows and ventilation points in a single African city,^69^ result in derived estimates for the proportion of remaining residual transmission exposure that occurs outdoors (assuming that a 90% protective effect of the screening is accounted for as previously described^9^) which are widely distributed across most of the full range of possible values.

However, many vector species exhibit considerable plasticity in these traits, so that each can adapt instantaneously and opportunistically to local, fine-scale heterogeneities in the availability of environmental resources. Many mosquito species have been observed to exhibit both extremes of human feeding versus animal feeding, indoor-feeding versus outdoor-feeding and indoor-resting versus outdoor-resting behaviours (figure 3). The ideal, but probably unachievable, optimal balance of vector control interventions can therefore vary greatly between neighbouring villages, or even within a single village. Of course, human beings are essential to malaria transmission, and also exhibit important plastic behavioural variations between individuals, families and communities that are driven by necessity, opportunities, culture and idiosyncrasy. Heterogeneities of mosquito and human behaviours (figure 3) create foci of low biological coverage of the blood and resting site resources targeted by each distinct vector control measure, bolstering malaria transmission against elimination with any single one of these intervention options. There is therefore no single theoretically ideal first-choice intervention: a combination will be required to deal with all extremes of this variation observed on fine geographic and demographic scales.

Fortunately, the extremes of variation in each behavioural phenotype that occur within the purview of any given control programme, which bolster transmission against any one of these interventions, also render it more vulnerable to the others. For example, while frequent feeding on animals in a subset of housing compounds within a single village (figure 3A) may attenuate the impact of insecticidal protection of humans using LLINs, IRS, insecticide-treated clothes or vapour-phase insecticide emanators, it also enables impact by insecticidal livestock (table 1), and the reverse may be true in a neighbouring compound where the same vector feeds mostly on humans (figure 3A). Similarly, while higher proportions of outdoor resting in different villages (figure 3B) can attenuate the local impact of IRS,^70^ and individual human tendencies to spend more time outdoors within a single city (figure 3C) can undermine the protective effects of mosquito-proofed housing,^69^ both phenomena should increase the impact of outdoor vapour-phase insecticides (table 1).

Of course, it is not realistic to monitor such behavioural metrics everywhere at all times across entire countries, so control programmes merely need sufficiently representative surveys to determine the range and distribution of values that intervention packages will need to address. The mean values obtained through such nationally representative or hot spot-targeted surveys may be used to prioritise front-line options in control programmes, while the extremes are indicative of what additional interventions may be required to eliminate malaria countrywide.

**Figure 4:**
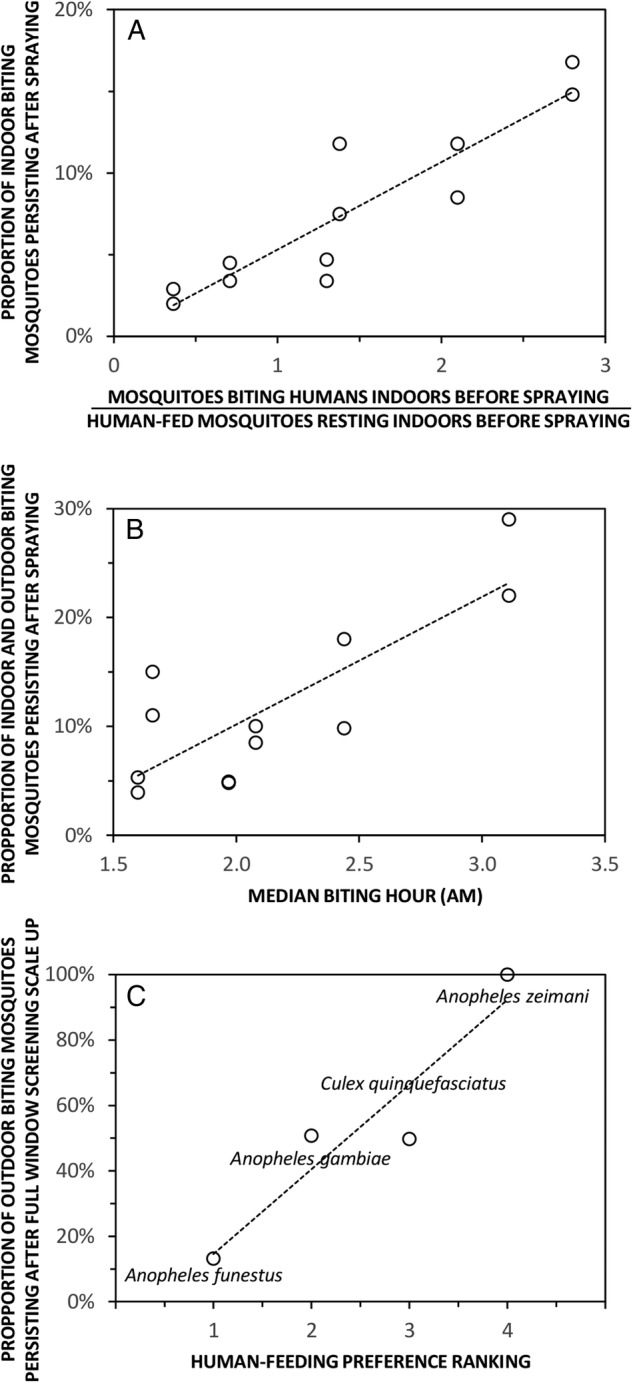
Examples of how field-surveyed metrics of adult mosquito behaviours can be predictive of vector control impact. The first two panels illustrate how preintervention measurements for indicators of outdoor resting (A) and early morning biting (B) were predictive of the impact of indoor residual spraying with Propoxur on *Anopheles gambiae sensu lato* during the Garki Project in northern Nigeria in the early 1970s,^70^ while the last panel illustrates how known preferences of mosquito species for feeding on humans are predictive of the impact of window screening in contemporary Dar es Salaam, coastal Tanzania (Chaki *et al*, Unpublished).

However, in order for control programme managers and product developers to confidently rely on such ‘cheap and cheerful' entomological indicators, they must first be rigorously evaluated across diverse settings in terms of their epidemiological predictive power. While the theoretical evidence base emphasising the importance of such behavioural measurements has become stronger in recent years,^3^ ^8^ ^52^ ^54^ ^55^ ^71–74^ direct empirical field assessments of their predictive value and generalisability are now urgently needed. To the best of our knowledge, no wide-scale, multisite assessment of the epidemiological relevance of any behavioural indicator other than the human blood index^3^ ^45^ ^46^ ^75^ has ever been conducted, but some examples from single-site studies give an encouraging idea of how this might be achieved and what kind of predictive values they may yield (figure 4).

### Restoring the problem-solving traditions of malaria vector surveillance

Developing and evaluating a simple set of affordable, practical, scalable entomological indicators of vector control opportunities will require considerable consensus and funding investment; it will also need a new generation of entomologists to embrace the quantitative ethos of what was once known as *epidemiological entomology*^76^ and update the underlying science. After decades of stagnation and excessive reliance on prescriptive global policies, it is high time to restore the historically creative traditions of malaria vector surveillance and control, which have yet to fully recover from the naïve adoption of IRS as a vector control panacea by the GMEP 60 years ago:

A serious consequence of that exaggerated confidence was the belief that the wide experience and knowledge of the old malariologists was superfluous and even counterproductive, particularly if they persisted in modifying the eradication strategy locally. Therefore, eradication campaigns were entrusted to new, preferably young ‘malariologists’, trained in ‘Malaria Eradication Training Centres’ established by WHO in several countries.^77^

Before DDT, malariologists were trained to be problem solvers; after DDT malariologists were trained to be solution implementers.^78^

The WHO has recently provided laudable leadership and direction by finally embracing a much more inclusive, devolved, diversified and adventurous, but nevertheless rational, approach to malaria vector control.^6^ This historic recent policy revision now encourages locally tailored, programmatic development of a much wider variety of malaria vector control interventions on a biologically rational basis.^6^ Those of us responsible for surveillance of malaria vector mosquito populations must now respond to this unprecedented formal broadening of our mandate. Sustainable entomological surveillance platforms are urgently needed that go beyond merely reporting physiological resistance to insecticides as the sole explanatory predictor of vector control impact. National and regional surveillance teams should now creatively and adaptively apply long neglected entomological techniques, to routinely measure targetable vector behaviours as a means to inform intervention choice and maximise impact.
